# Analysis of Peripheral Blood IL-6 and Leukocyte Characteristics in 364 COVID-19 Patients of Wuhan

**DOI:** 10.3389/fimmu.2020.559716

**Published:** 2020-11-19

**Authors:** Quan Li, Yuyu Xie, Zhangbo Cui, Shi Tang, Baodong Yuan, Hai Huang, Yanjie Hu, Yaling Wang, Ming Zhou, Chunwei Shi

**Affiliations:** ^1^ Department of Tuberculosis Ward, Wuhan Pulmonary Hospital , Wuhan, China; ^2^ Department of Pathogen Biology, School of Basic Medicine, Tongji Medical College, Huazhong University of Science and Technology, Wuhan, China; ^3^ Department of Clinical Laboratory, Wuhan Pulmonary Hospital, Wuhan, China; ^4^ Department of Pharmacy, Wuhan Pulmonary Hospital, Wuhan, China

**Keywords:** 2019 coronavirus disease, SARS coronavirus 2, IL-6, leukocytes, lymphocytes, neutrophils, monocytes

## Abstract

SARS-CoV-2, the pathogen of COVID-19, is spreading around the world. Different individuals infected with COVID-19 have different manifestations. It is urgent to determine the risk factors of disease progress of COVID-19. 364 patients diagnosed with COVID-19, who were admitted to Wuhan Pulmonary Hospital from February 3, 2020 to March 16, 2020, were divided into mild, ordinary, severe, and critical groups, according to Chinese novel coronavirus pneumonia diagnosis and treatment plan. Peripheral blood IL-6 and leukocyte characteristics were analyzed, to evaluate the correlation with the severity of COVID-19. The levels of peripheral blood IL-6 were 2.35 ± 0.46 pg/ml (mild), 6.48 ± 1.13 pg/ml (ordinary), 20.30 ± 5.15 pg/ml (severe), and 123.48 ± 44.31 pg/ml (critical). The leukocytes were 5.70 ± 0.41×10^9^/L (mild), 6.21 ± 0.14×10^9^/L (ordinary), 6.37 ± 0.26×10^9^/L (severe), and 10.03 ± 1.43×10^9^/L (critical). The lymphocytes were 1.46 ± 0.19×10^9^/L (mild), 1.89 ± 0.14×10^9^/L (ordinary), 1.26 ± 0.07×10^9^/L (severe), and 1.17 ± 0.23×10^9^/L (critical). The neutrophils were 3.63 ± 0.36×10^9^/L (mild), 3.78 ± 0.11×10^9^/L (ordinary), 4.47 ± 0.25×10^9^/L (severe), and 7.92 ± 1.19×10^9^/L (critical). The monocytes were 0.42 ± 0.05×10^9^/L (mild), 0.44 ± 0.01×10^9^/L (ordinary), 0.46 ± 0.02×10^9^/L (severe), and 0.78 ± 0.25×10^9^/L (critical). Conclusively, increase of peripheral blood IL-6 and decrease of lymphocytes can be used as the indicators of severe COVID-19. The increase of neutrophils and monocytes was noticed in critical cases of COVID-19, suggesting that the increase of neutrophils and monocytes should be considered as risk factors of critical cases of COVID-19. Peripheral blood IL-6 and leukocyte characteristics were also analyzed in different age groups. The increase of serum IL-6, decrease of lymphocytes, and increase of neutrophils were noticed in patients over 60 years old.

## Introduction 

Since the outbreak of 2019 coronavirus disease (COVID-19) in Wuhan at the end of December 2019, the epidemic has spread rapidly, with confirmed and fatal cases in many countries around the world. As of April 19, 2020, a total of 2,241,778 cases have been confirmed globally, with a total of 152,551 deaths (1). The pathogen causing COVID-19 was a novel coronavirus (2019 novel coronavirus, 2019nCoV) (2), lately named as SARS coronavirus 2 (SARS-CoV-2) (3), which was confirmed by viral genome sequencing in alveolar lavage fluid of three COVID-19 patients in Jinyintan Hospital, Wuhan city (2). SARS-CoV-2 is a betacoronavirus and the genome is positive single-stranded RNA, with an envelope on which the mushroom protein spines make the virus shape like a crown (2).

Different individuals infected with COVID-19 have different manifestations. It is urgent to determine the risk factors of disease progress of COVID-19. Further research is being carried out on the pathogenesis of COVID-19 caused by SARS-CoV-2 infection. According to the latest research findings, as humans are infected with SARS-CoV-2, CD4^+^ T cells are rapidly activated, proliferate and differentiate into Th1 cells, and produce granulocyte-macrophage colony stimulating factor (GM-CSF), which further induces inflammatory CD14^+^CD16^+^ monocytes to over-express IL-6 and other factors, promoting inflammatory responses (4). It is speculated that GM-CSF and IL-6 may be the key factors inducing cytokine storm. In addition, peripheral lymphocytopenia in patients with COVID-19 was revealed by some retrospective studies (5–8). Therefore, we suspected that in the pathogenesis of COVID-19, both lymphocyte injury and cytokine storm might be involved. Three hundred sixty-four patients diagnosed with COVID-19, who were admitted to Wuhan Pulmonary Hospital from February 3, 2020 to March 16, 2020, were divided into mild, ordinary, severe, and critical groups, according to Chinese novel coronavirus pneumonia diagnosis and treatment plan. Peripheral blood IL-6 and leukocyte characteristics were retrospectively analyzed, to evaluate the correlation of these laboratory indicators with the severity of COVID-19.

## Materials and Methods

### Patients

Three hundred sixty-four patients diagnosed with COVID-19, who were admitted to Wuhan Pulmonary Hospital from February 3, 2020 to March 16, 2020, were divided into mild, ordinary, severe, and critical groups, according to Chinese novel coronavirus pneumonia diagnosis and treatment plan: mild type, with slight clinical symptoms but no imaging presentation of pneumonia; ordinary type, with fever, respiratory tract and other symptoms, imaging findings of pneumonia; severe type, with any of the following conditions: respiratory distress, respiratory frequency ≥30 times/minutes, finger oxygen saturation at rest ≤93%, or oxygenation index [PaO2/FiO2] ≤300 mmHg (1 mmHg = 0.133 kPa); critical type, with any of the following conditions: respiratory failure requires mechanical ventilation, shock, combined with other organ failure that requires intensive care unit care and treatment (8). All the patients were diagnosed through the positive detection of SARS-CoV-2 nucleic acid from throat swabs or sputum samples.

### Detection of SARS-CoV-2 Nucleic Acid

Samples of pharyngeal swabs and sputum from patients were collected. RNA was extracted using RNA extraction kit (2020031, Da ‘an, China) and Real-Time PCR was performed to detect the sequence of N protein and ORF1ab of SARS-CoV-2 using quantitative PCR kit (DA0930, Da ‘an, China) with Real-Time PCR apparatus (7500, ABI, USA).

### Routine Blood Test

2 ml of venous blood of patients was collected in the EDTA-2K anti-coagulant tube and routine blood test was detected (BC-6900, Mindray, China). The data of routine blood test collected in this study were all the analysis of the first test results after admission.

### Detection of Serum IL-6

The peripheral blood of patients was collected and centrifuged at 4,000 rpm for 4 min. The serum concentration of IL-6 was measured (Cobas 8000, Roche, USA). All the data of IL-6 collected in this study were the first detection after the patient was admitted to the hospital.

### Statistical Analysis

Categorical variables were described as percentages, continuous variables were described as mean, standard errors of the mean, and interquartile range (IQR) values. A nonparametric test (kruskal-wallis rank sum and unpaired, two-sided Mann–Whitney U-test) was used to compare the means of continuous variables. All statistical analyses were performed using SPSS 19.0 software (SPSS Inc). *P <*0.05 was considered statistically significant.

## Results

### Patients’ Characteristics

**Figure 1:**
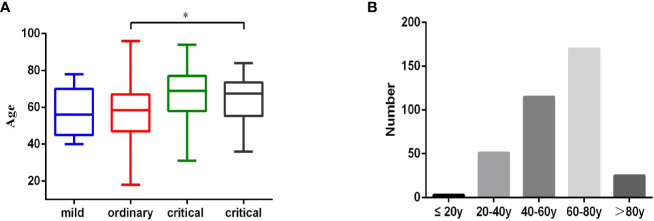
Age characteristics of 364 COVID-19 patient. **(A)**. 364 patients diagnosed with COVID-19, who were admitted to Wuhan Pulmonary Hospital from February 3, 2020 to March 16, 2020, were divided into mild (n = 11), ordinary (n = 268), severe (n = 67), and critical (n = 18) groups according to Chinese novel coronavirus pneumonia diagnosis and treatment plan. Age information of the patients in each group was analyzed. Statistical analysis was performed using a nonparametric test (kruskal-wallis rank sum) and data of age were expressed as mean (IQR), *p < 0.05. **(B)**. The age distribution of all 364 patients.

Three hundred sixty-four patients diagnosed with COVID-19, who were admitted to Wuhan Pulmonary Hospital from February 3, 2020 to March 16, 2020, were divided into mild, ordinary, severe and critical groups, according to Chinese novel coronavirus pneumonia diagnosis and treatment plan. All the patients were diagnosed through the positive detection of SARS-CoV-2 nucleic acid from throat swabs or sputum samples. The average age of the 364 patients is 62 years old. In the mild group, there were 11 cases (3.02%), with an average age of 56 years (IQR, 45–70, range, 40–78 years). In the ordinary group, there were 268 cases (73.63%), with an average age of 58.5 years (IQR, 47–67, range 18–96 years). There were 67 patients (18.40%) in the severe group, with an average age of 69 years (IQR, 58–77, range of 31–94 years). There were 18 patients (4.95%) in the critical group, with an average age of 67.5 years (IQR, 55.25–73.5, range of 36–84 years) (**Table 1** and **Figure 1A**). Age distribution of the patients was analyzed. There were 3 patients under the age of 20 years (0.82%), 51 patients between the ages of 20 and 40 years (14.01%), 115 patients between the ages of 40 and 60 years (31.59%), 170 patients between the ages of 60 and 80 years (46.70%), and 25 patients over the age of 80 years (6.86%) (**Figure 1B** and **Table 4**). Similar to other retrospective studies, COVID-19 patients admitted to Wuhan Pulmonary Hospital had the largest number of patients in the ordinary group, followed by patients in the severe group, and fewer patients in mild and critical groups (9, 10).

**Table 1:** Patient characteristics.

	Total (N = 364)	Mild (N = 11)	Ordinary (N = 268)	Severe (N = 67)	Critical (N = 18)
**Age, median (IQR)**	62 (49–69.75)	56 (45–70)	58.5 (47–67)	69 (58–77)	67.5 (55.25–73.50)
**Sex**					
**Female (%)**	203 (56)	6 (55)	156 (58)	34 (51)	7 (39)
**Male (%)**	161 (44)	5 (45)	112 (42)	33 (49)	11 (61)

Three hundred sixty-four patients diagnosed with COVID-19, who were admitted to Wuhan Pulmonary Hospital from February 3, 2020 to March 16, 2020, were divided into mild, ordinary, severe, and critical groups, according to Chinese novel coronavirus pneumonia diagnosis and treatment plan. Information of the patients including age and gender in each group was analyzed. Statistical analysis was performed and data of age were expressed as mean (IQR).

### Characteristics of IL-6 in Peripheral Blood of Patients With COVID-19

**Figure 2:**
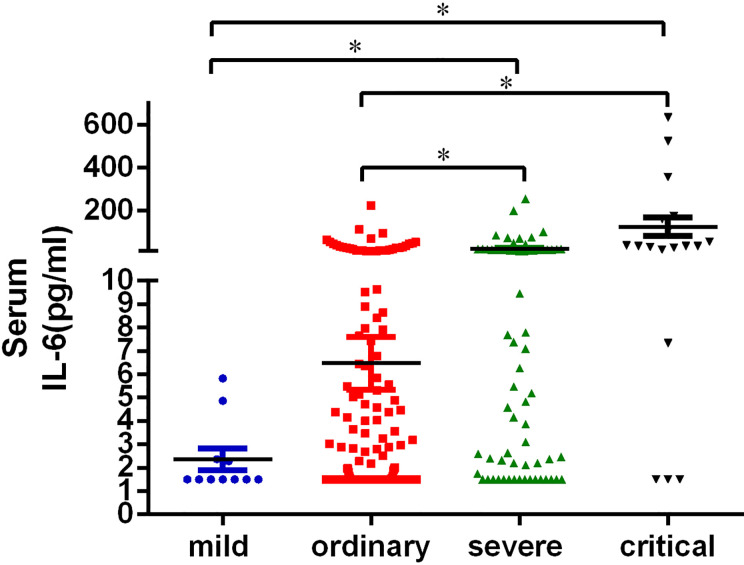
Serum IL-6 level in patients with COVID-19. The peripheral blood of patients was collected and centrifuged at 4,000 rpm for 4min. The serum concentration of IL-6 was measured. All the data of IL-6 collected in this study were the first detection after the patient was admitted to the hospital. Statistical analysis of the serum IL-6 of the patients in mild (n = 11), ordinary (n = 268), severe (n = 67), and critical (n = 18) groups was performed using unpaired, two-sided Mann-Whitney U-test and data were expressed as mean ± SEM, *p < 0.05.

The reference value of IL-6 is 0–7 pg/ml. Increase of serum IL-6 was not observed in mild patients. The percentage of patients with increased serum IL-6 was 15.30% in the ordinary group, 49.25% in the severe group and 83.33% in the critical group (**Table 2**). The concentration of serum IL-6 of mild, ordinary, severe, and critical patients was 2.35 ± 0.46 pg/ml, 6.48 ± 1.13 pg/ml, 20.30 ± 5.15 pg/ml, and 123.48 ± 44.31 pg/ml, respectively. Among them, the increase of IL-6 in severe and critical patients was significant, as compared with mild and ordinary patients (*P* < 0.05) (**Figure 2**). Conclusively, increase of serum IL-6 is common in severe and critical forms of COVID-19, especially in critical cases.

**Table 2:** Characteristics of serum IL-6 in patients with COVID-19.

		mild group	ordinary group	severe group	critical group
**IL-6**	within normal values (n,%)	11(100.00)	227(84.70)	34(50.75)	3(16.67)
	above normal values (n,%)	–	41(15.30)	33(49.25)	15(83.33)

The peripheral blood of patients was collected and centrifuged at 4,000 rpm for 4min. The serum concentration of IL-6 was measured. All the data of IL-6 collected in this study were the first detection after the patient was admitted to the hospital. The percentage of patients with elevated IL-6 was calculated in mild, ordinary, severe, and critical groups, according to the reference of serum IL-6 concentration (0–7 pg/ml).

### Characteristics of Peripheral Blood Leukocytes in Patients With COVID-19

**Figure 3:**
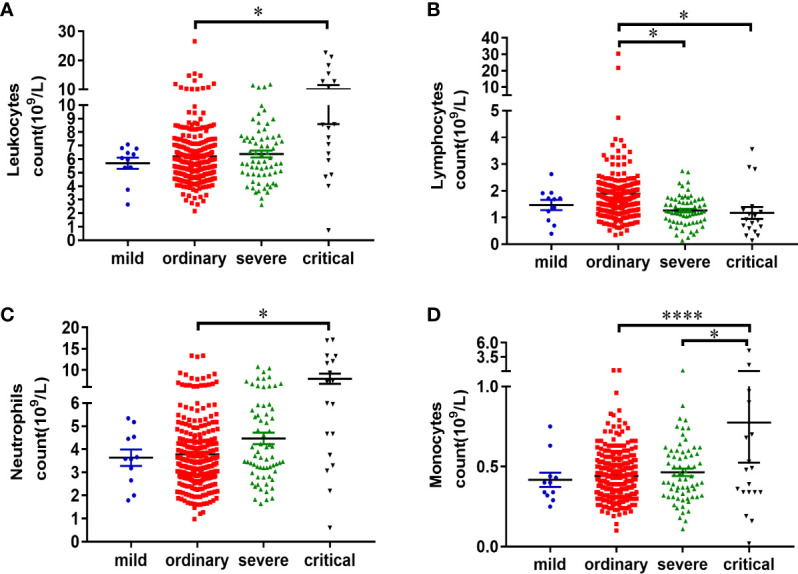
Characteristics of peripheral blood leukocytes in patients with COVID-19. Information of the routine blood test of patients was analyzed. All the data of routine blood test collected in this study were the first detection after the patient was admitted to the hospital. Statistical analysis of the leukocyte count **(A)**, lymphocyte count **(B)**, neutrophil count **(C)**, and monocyte count **(D)** of the patients in mild (n = 11), ordinary (n = 268), severe (n = 67), and critical (n = 18) groups was performed using unpaired, two-sided Mann-Whitney U-test and data were expressed as mean ± SEM, *p < 0.05, ****p < 0.0001.

The reference value of leukocytes is 3.5–9.5×10^9^/L. According to the blood count of the patients, over 90% of patients in the mild and ordinary groups, had the normal leukocyte count. In the severe group, 88.06% patients had the normal leukocyte count. However, in the critical group, leukocyte count was normal in only 55.56% patients, while 38.89% patients had the increased leukocyte count and 5.56% patients had the decreased leukocyte count (**Table 3**). The mean leukocyte count of the patients in the mild, ordinary, severe and critical groups was 5.70 ± 0.41×10^9^/L, 6.21 ± 0.14×10^9^/L, 6.37 ± 0.26×10^9^/L, and 10.03 ± 1.43×10^9^/L, respectively (**Figure 3A**). The increase of leukocyte count in critical patients was significant, as compared with ordinary patients (*P* < 0.05) (**Figure 3A**).

**Table 3:** Characteristics of peripheral blood leukocytes in patients with COVID-19.

		mild group	ordinary group	severe group	critical group
**Leukocyte count**	below normal values (n,%)	1(9.09)	10(3.73)	2(2.99)	1(5.56)
	within normal values (n,%)	10(90.91)	244(91.04)	59(88.06)	10 (55.56)
	above normal values (n,%)	–	14(5.22)	6 (8.96)	7(38.89)
**Lymphocyte count**	below normal values (n,%)	3(27.27)	40(14.93)	24(35.82)	11(61.11)
	within normal values (n,%)	8(72.73)	215(80.22)	43(64.18)	6(33.33)
	above normal values (n,%)	–	13(4.85)	–	1(5.56)
**Neutrophil count**	below normal values (n,%)	1(9.09)	14(5.22)	1(1.50)	1(5.56)
	within normal values (n,%)	10(90.91)	235(87.69)	56(83.58)	8(44.44)
	above normal values (n,%)	–	19(7.09)	10(14.93)	9(50.00)
**Monocyte count**	below normal values (n,%)	–	–	–	1(5.56)
	within normal values (n,%)	9(81.82)	234(98.73)	54(80.60)	11(61.11)
	above normal values (n,%)	2(18.18)	33(13.92)	13(19.40)	6(33.33)

Information of the routine blood test of patients in mild, ordinary, severe, and critical groups was analyzed. All the data of routine blood test collected in this study were the first detection after the patient was admitted to the hospital. The percentage of patients with abnormal leukocyte count, lymphocyte count, neutrophil count, and monocyte count was calculated in each group, according to the reference (leukocyte count: 3.5–9.5×10^9^/L, lymphocyte count: 1.1–3.2×10^9^/L, neutrophil count: 1.8–6.3×10^9^/L, monocyte count: 0.1–0.6×10^9^/L).

The count of lymphocytes, neutrophils, and monocytes was further analyzed. The reference count of lymphocytes is 1.1–3.2×10^9^/L. According to the results, 27.27% patients in the mild group, 14.93% patients in the ordinary group, 35.82% patients in the severe group, and 61.11% patients in the critical group, had decreased lymphocyte count (**Table 3**). The lymphocyte counts were 1.46 ± 0.19×10^9^/L in the mild patients, 1.89 ± 0.14×10^9^/L in the ordinary patients, 1.26 ± 0.07×10^9^/L in the severe patients, and 1.17 ± 0.23×10^9^/L in the critical patients, respectively. There was a significant decrease of lymphocytes in the severe and critical groups, as compared with ordinary group (*P* < 0.05) (**Figure 3B**).

The reference count of neutrophils is 1.8–6.3×10^9^/L. No increase of neutrophils was observed in the mild group. While 7.09% patients in the ordinary group, 14.93% patients in the severe group, 50.00% patients in the critical group, had the increased neutrophils (**Table 3**). The count of neutrophils was respectively 3.63 ± 0.36×10^9^/L in the mild patients, 3.78 ± 0.11×10^9^/L in the ordinary group, 4.47 ± 0.25×10^9^/L in the severe group, and 7.92 ± 1.19×10^9^/L in the critical group (**Figure 3C**). The increase of neutrophil count in critical patients was significant, as compared with ordinary patients (*P* < 0.05) (**Figure 3C**).

The reference count of monocytes is 0.1–0.6×10^9^/L. According to the results, 18.18% patients in the mild group, 13.92% patients in the ordinary group, 19.40% patients in the severe group, and 33.33% patients in the critical group, had increased monocyte count (**Table 3**). The count of monocytes was respectively 0.42 ± 0.05×10^9^/L in the mild patients, 0.44 ± 0.01×10^9^/L in the ordinary group, 0.46 ± 0.02×10^9^/L in the severe group, and 0.78 ± 0.25×10^9^/L in the critical group (**Figure 3D**). There was a significant increase of monocytes in the critical group, as compared with ordinary and severe groups (*P* < 0.05) (**Figure 3D**). Taken together, lymphocyte decrease and neutrophil increase are common leukocyte characteristics in critical cases of COVID-19. In addition, increase of monocytes was observed in one third of critically ill patients.

### Analysis of Serum IL-6 and Peripheral Blood Leukocyte Characteristics in Different Age Groups

**Figure 4:**
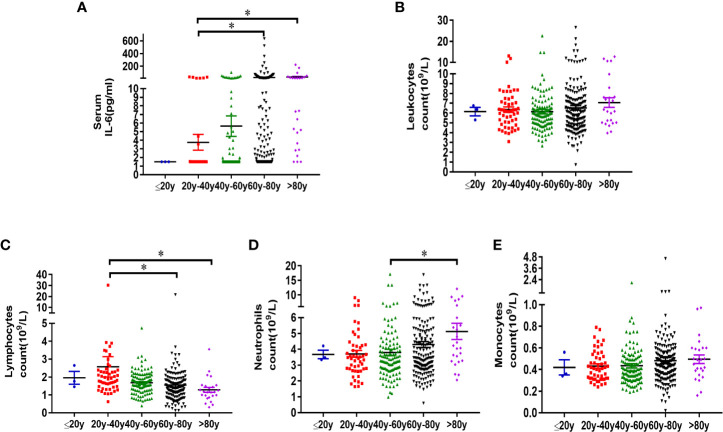
Analysis of serum IL-6 and peripheral blood leukocyte characteristics in different age groups. Three hundred sixty-four patients diagnosed with COVID-19, who were admitted to Wuhan Pulmonary Hospital from February 3, 2020 to March 16, 2020, were divided into different age groups (Age ≤ 20, n = 3; 20 < Age ≤ 40, n = 51; 40 < Age ≤ 60, n = 115; 60 < Age ≤ 80, n = 170; Age > 80, n = 25). Information of serum IL-6 (a), leukocyte count **(B)**, lymphocyte count **(C)**, neutrophil count **(D)** and monocyte count **(E)** of patients in each group was analyzed. Statistical analysis was performed using unpaired, two-sided Mann-Whitney U-test and data were expressed as mean ± SEM, *p < 0.05.

Peripheral blood IL-6 and leukocyte characteristics were also analyzed in different age groups. The increase of serum IL-6, decrease of lymphocytes were also noticed in patients over 60 years old. The concentration of serum IL-6 in different age group was 1.50 ± 0.00 pg/ml (≤ 20y), 3.76 ± 0.92 pg/ml (20 < Age ≤ 40), 5.65 ± 1.19 pg/ml (40 < Age ≤ 60), 22.16 ± 5.66 pg/ml (60 < Age ≤ 80), and 29.29 ± 11.04 pg/ml (Age > 80), respectively (**Table 4**). Since there were only 3 patients under the age of 20 years, statistical analysis was performed to compare the difference of other age groups with the group between the ages of 20 and 40 years. The level of serum IL-6 increased significantly in elderly patients over 60 years old, as compared with 20–40-year patients (p < 0.05) (**Figure 4A**). No difference of leukocyte count was observed in different age groups (**Figure 4B**). Significant decrease of lymphocytes was observed in groups over 60 years old, as compared with 20–40-year group (p < 0.05) (**Figure 4C**). Increase of neutrophils was only observed in the group over 80 years old (**Figure 4D**). No difference of monocyte count was observed in different age groups (**Figure 4E**). Conclusively, the increase of serum IL-6, decrease of lymphocytes and increase of neutrophils should be also paid attention in older patients infected of COVID-19.

**Table 4:** Analysis of serum IL-6 and peripheral blood leukocyte characteristics in different age groups.

	Age ≤ 20	20＜Age ≤ 40	40＜Age ≤ 60	60＜Age ≤ 80	Age＞80
**Patiens Number**	3(0.82%)	51(14.01%)	115(31.59%)	170 (46.70%)	25(6.86%)
**Serum IL-6 (pg/ml)**	1.50 ± 0.00	3.76 ± 0.92	5.65 ± 1.19	22.16 ± 5.66	29.29 ± 11.04
**Leukocyte count (10^9^/L)**	6.14 ± 0.43	6.35 ± 0.27	6.14 ± 0.22	6.52 ± 0.24	7.06 ± 0.49
**Lymphocyte count (10^9^/L)**	1.96 ± 0.36	2.57 ± 0.57	1.68 ± 0.06	1.56 ± 0.13	1.28 ± 0.14
**Neutrophil count (10^9^/L)**	3.67 ± 0.27	3.71 ± 0.21	3.80 ± 0.20	4.30 ± 0.19	5.13 ± 0.51
**Monocyte count (10^9^/L)**	0.42 ± 0.07	0.43 ± 0.02	0.43 ± 0.02	0.48 ± 0.03	0.49 ± 0.04

Three hundred sixty-four patients diagnosed with COVID-19, who were admitted to Wuhan Pulmonary Hospital from February 3, 2020 to March 16, 2020, were divided into different age groups (Age ≤ 20, 20＜Age ≤ 40, 40＜Age ≤ 60, 60＜Age ≤ 80, Age＞80). Information of serum IL-6 and the routine blood test of patients in each group was analyzed. Statistical analysis was performed and data were expressed as mean ± SEM.

## Discussion

A total of 364 patients diagnosed with COVID-19, who were admitted to Wuhan Pulmonary Hospital from February 3, 2020 to March 16, 2020, were included in this study and divided into mild, ordinary, severe and critical forms, according to Chinese novel coronavirus pneumonia diagnosis and treatment plan. It was reported that most of the patients infected with COVID-19 were mild cases. However, Wuhan Pulmonary Hospital was a designated hospital for COVID-19 treatment and mainly received patients of non-mild form. Therefore, COVID-19 patients in this study, had the largest number of patients in the ordinary group (73.63%), followed by patients in the severe group (18.40%), and fewer patients in mild (3.02%) and critical (4.95%) groups, similar to other retrospective studies (9, 10).

It was observed that serum IL-6 level was not significantly increased in patients in mild and ordinary groups without significant lung injury indicated by lung CT. However, 49.25% of patients in the severe group and 83.33% of patients in the critical group had the increased serum IL-6, indicating the severity of the disease is accompanied by inflammatory reaction. In current study, the increase of leukocyte count and neutrophil count was also observed, especially in critically ill patients. It has been recently reported that the counts of leukocytes and neutrophils of COVID-19 victims were higher than those of the survivors (11). Increase of neutrophils might be related with the secondary bacterial infection due to immunosuppression caused by virus infection. We also suspected that neutrophils might be involved in inflammation of COVID-19 pathogenesis, since some patients with COVID-19 had obvious increase of neutrophils in the early stage of the disease, without apparent sign of bacterial infection. Recently, it was reported that neutrophils played an important role in inflammatory reaction (12, 13). Upon COVID-19 infection, neutrophils released higher levels of neutrophil extracellular traps (NETs), which act as important mediators of tissue damage in inflammatory reaction (14).

Circulating monocytes in peripheral blood express a broad repertoire of plasma membrane and intracellular receptors, serving as sensors of micro-organisms, dying cells and soluble products, to mediate innate immune response. Monocytes differentiate into resident macrophages, following tissue inflammation, infection, or malignancy. Monocyte-derived macrophages are reported to be the main generators of inflammation in COVID-19 (15, 16). In this study, the increase of monocytes was noticed in critical group. The increase of neutrophils and monocytes, two kinds of important cells in innate immunity, indicated the activation of innate immunity and the inflammatory reaction, which promotes the elimination of virus by adaptive immune mechanism.

However, lymphocytopenia is a common feature in critically ill patients. In the critical group, 61.11% of the patients had decreased lymphocytes, while in the severe group, about one third of the patients had decreased lymphocyte count. A number of retrospective studies have reported that lymphocytopenia is common in COVID-19 patients (5–8) and lymphocytopenia can be regarded as an indicator of the progression of COVID-19 (17). At present, there is no clear understanding about the mechanism of lymphocytopenia in COVID-19. Some studies have pointed out that host factors may be involved in the reduction of lymphocytes (18). It was reported that the patients with severe and critical COVID-19 infection are generally older, as compared with patients with mild and ordinary form, and have a higher proportion of underlying diseases such as hypertension, diabetes, cardiovascular or cerebrovascular diseases (10). From the analysis of the patients’ information in this study, we have also observed significant decrease of lymphocytes in groups over 60 years old. Aging and chronic diseases are more likely to lead to chronic endothelial dysfunction, which causes the destruction of the cell-cell connection, death of endothelial cells, destruction of the blood-tissue barriers, enhancement of leukocyte adhesion and exudation (13), which may help us explain the lymphopenia in peripheral circulation in elderly patients, in severe and critical COVID-19 cases. Increase of neutrophils and monocytes, along with decreased lymphocytes in critically ill patients of COVID-19, demonstrated dysregulation of innate and acquired immunity. The detailed mechanisms, why activated innate immunity cannot effectively promote the acquired immunity to clear the virus, but cause immunopathology, including lymphocytopenia, inflammatory tissue damage, should be further researched in the pathogenesis of COVID-19.

Different individuals infected with COVID-19 have different manifestations and different outcomes. With the spread of SARS-CoV-2 in many countries around the world, it is urgent to determine the criteria for severe diseases, especially critical cases. Detection of inflammatory cytokines represented by serum IL-6 and routine blood tests can be quickly and easily performed. Through the study, we found that increased serum IL-6, decreased lymphocytes, and increased neutrophils and monocytes, can help us to assess critical patients with poor clinical outcomes.

Conclusively, this retrospective study analyzed the correlation of serum IL-6 and leukocyte characteristics with the severity of COVID-19. Peripheral blood IL-6 and leukocyte characteristics were also analyzed in different age groups. The increase of serum IL-6, decrease of lymphocytes, and increase of neutrophils were also noticed in patients over 60 years old, which help us understand why COVID-19 is more dangerous for the elder population.

## Data Availability Statement

The raw data supporting the conclusions of this article will be made available by the authors, without undue reservation.

## Ethics Statement

The studies involving human participants were reviewed and approved by the institutional review board of Wuhan pulmonary hospital. The patients/participants provided their written informed consent to participate in this study.

## Author Contributions

QL, MZ, BY, HH, YH, and YW performed the experiments in this study. QL, CZ, ST, and YX analyzed and interpreted the data. CZ performed statistical analysis. QL, YX, MZ, and CS wrote the manuscript. All authors contributed to the article and approved the submitted version.

## Funding

This work was supported by grants of Science and Technology Project of Wuhan City (WX16C21) and Wuhan Health Research Fund (EX20E11).

## Conflict of Interest

The authors declare that the research was conducted in the absence of any commercial or financial relationships that could be construed as a potential conflict of interest.
